# Gene Banks, Seed Libraries, and Vegetable Sanctuaries: The Cultivation and Conservation of Heritage Vegetables in Britain, 1970–1985

**DOI:** 10.1111/cuag.12239

**Published:** 2019-11-26

**Authors:** Helen Anne Curry

**Keywords:** seed saving, gene bank, crop genetic diversity, Henry Doubleday Research Association, Garden Organic, Lawrence Hills

## Abstract

Individual seed saving and exchange are considered important components of contemporary efforts to conserve crop genetic diversity that ramify at local, regional, and global scales. Yet the very fact that the contributions of these activities to conservation need to be made explicit by seed savers and those who study them indicates that the practices of seed saving and exchange may not immediately be recognized as conservation‐oriented activities. This article investigates why and how individual seed saving came to be aligned with a broader conservation agenda in Britain through a historical examination of the promotion of seed saving by the Henry Doubleday Research Association (HDRA) in the 1970s and 1980s. It demonstrates how several HDRA initiatives that aimed to preserve vegetable diversity also re‐inscribed British gardeners’ ordinary labor as conservation work. This historical study complements sociological and ethnographic studies, highlighting the role of a prominent organization in creating pathways for individuals to engage in local, national, and international conservation through seed saving. It also serves as a reminder that the connections between these activities had to be made explicit—that is, that there was (and is) work involved in connecting individual acts of seed saving to conservation outcomes at different scales.

In 2018, the British charity Garden Organic, an organization dedicated to fostering the use of organic methods of cultivation, explained the work of its Heritage Seed Library to website visitors. The library includes about 800 different vegetable varieties, most of which are heirlooms, European landraces, or retired commercial lines and therefore not widely available. According to the website description, by raising vegetables using seed from the library, gardeners can increase the diversity of plants in their gardens and save their own seed from year to year. Through these actions, they can also support “the conservation of unusual vegetable varieties for future generations” and help “maintain genetic diversity within vegetable crops” that might be needed by plant breeders (Garden Organic 2018). This conjunction of near‐term personal goals with longer‐term conservation interests, and the achievement of both through the typical farm and garden activities of saving, exchanging, and cultivating seeds, appears frequently in the literature produced by seed saving organizations. The prominent US network Seed Savers Exchange declares that its mission is “to conserve and promote America's culturally diverse but endangered garden and food crop heritage for future generations by collecting, growing, and sharing heirloom seeds and plants” (Seed Savers Exchange 2018; see also Whealy 2011).

The confluence of near‐term individual goals with long‐term community interests also appears frequently in the literature *about* these organizations and the seed savers they champion. In recent years, a number of scholars have documented the work of seed savers (by which I mean individuals and organizations engaged in the cultivation and local exchange of seeds of so‐called traditional, heritage, or heirloom varieties) in several countries and across different socioeconomic contexts.^1^ A recurring theme in this literature is the role played by seed savers in conserving the genetic diversity of agricultural crops. Researchers have sought to establish the direct contributions of seed savers to conservation, for example, in charting the nature and extent of the crop diversity perpetuated through their activities (Ellen and Platten 2011; Veteto 2007, 2014); the ways in which their conservation targets differ from those of state institutions (Carolan 2006; Van Dooren 2009); and the other conservation‐related benefits of their activities such as increasing awareness about biodiversity (Steinberg 2001), renewing agrarian knowledge networks (Campbell 2012), and generating more resilient food systems (Helicke 2015). Anthropologists, ethnographers, and geographers have developed rich accounts of what prompts individuals to want to conserve crop diversity (especially Nazarea 2005; see also Carolan 2007; Jordan 2010; Purdue 2000), including assessments of their perspectives on seed saving as a politically engaged activity (Phillips 2008; Pottinger 2017a,b).

This body of research provides a persuasive picture of individual seed saving and exchange as important components of contemporary efforts to conserve and promote crop genetic diversity, the effects of which ramify at local, regional, and global scales. Yet the very fact that the contributions of these practices to conservation need to be made explicit by both seed savers and those who study them indicates that seed saving and exchange may not immediately be recognized as conservation‐oriented activities. These are two foundational acts of agricultural production, and they continue to be routine endeavors for cultivators in many parts of the world. By comparison, in places where agricultural industrialization, the growth of commercial seed industry, and the effects of intellectual property protection have distanced consumers from farming and farmers from seed production (Fitzgerald 1990; Kloppenburg 2004; Mascarenhas and Busch 2006), these activities have sometimes had to be rediscovered by late 20th‐century growers. This rediscovery was and is tied up with varied agendas, but especially with the search for alternatives to industrial food production and in some cases industrial society as a whole (see examples in Helicke 2015; Phillips 2013; Steinberg 2001). From the 1970s onward, it was additionally informed by an emerging international consensus about the loss of genetic diversity in crops resulting from global agricultural change (Fenzi and Bonneuil 2016; Pistorius 1997; Pistorius and Van Wijk 1999). In this context, seed savers and especially the organizations that endeavored (and still endeavor) to bring them together forged a new understanding of seed saving as a practice that not only secures alternative agricultural trajectories in the present by preserving diverse crop varieties, but also ensures these that these possibilities will endure in the future.

In what follows, I contribute to the literature on seed saving by exploring in greater detail why and how individual seed saving came to be aligned with a broader conservation agenda. Through a historical examination of the activities of the Henry Doubleday Research Association (HDRA), the forerunner of today's Garden Organic, I follow the development of a new discourse around seed saving that targeted British gardeners. I argue that several HDRA initiatives that aimed to preserve vegetable varieties useful to and desired by “own‐growers” in the 1970s and 1980s, including the creation of what would become its Heritage Seed Library, also re‐inscribed gardeners’ ordinary labor as conservation work.^2^ The trajectories of these initiatives are documented in HDRA books, pamphlets, and newsletters, as well as historical newspaper articles and more recent recollections.^3^ Drawing on these materials, I explore the initial motivations of HDRA and its tireless director Lawrence Hills in encouraging seed saving among British and other own‐growers. I detail the activities they recommended to gardeners as ways of contributing to the perpetuation of threatened vegetable varieties and how Hills and HDRA situated these in relation to national and international activities. Throughout, I suggest how the initiatives spearheaded by HDRA worked to imbue individual gardening decisions with global conservation significance.

This historical work complements sociological and ethnographic studies, especially those that have followed HDRA and Heritage Seed Library subscribers (Pottinger 2017a,b; Purdue 2000) and other British seed savers (Gilbert 2013). First, it highlights the role of a prominent organization in creating new ways for the subjects of these existing studies to engage in local, national, and international conservation through seed saving. Second, it serves as a reminder that the connections between these activities had to be made explicit—that is, that there was (and is) work involved in connecting individual acts of seed saving to conservation outcomes at different scales.

## Endangered Vegetables

The British writer and horticulturist Lawrence Hills founded HDRA in Essex, England, in 1954 to encourage gardeners to experiment independently with organic methods of cultivation (Martin 2011). With Hills at the helm, HDRA organized investigations into the shared concerns of its members and other gardeners and pooled their experiences and observations through a regular newsletter. HDRA also published pamphlets and booklets on subjects such as natural fertilizers, pesticide alternatives, and varietal recommendations, which it typically disseminated free to its members and sold for a small fee to non‐members. Hills was the chief energetic force behind HDRA in its first two decades and more, authoring the quarterly newsletter and many of its publications, spearheading talks and displays, leading tours of the HDRA trial grounds, and visiting HDRA affiliates abroad. In fact, Hills was so central to HDRA activities through the 1970s that it is often difficult to distinguish his voice from that of the organization. Despite Hills's influence, HDRA was not a one‐man show. He recruited a slate of officers to govern the organization, eventually brought on paid staff, and encouraged an active membership whose views he regularly aired in the HDRA newsletter.^4^ HDRA existed primarily to aggregate the knowledge of its members, the number of which grew steadily from 100 in 1958 to about 1,200 in 1964. By the early 1970s, when membership cost £3 per year, HDRA claimed some 3,000 members scattered across a number of countries, including the United States (U.S.), Canada, India, and Australia.

The investigation and promotion of methods of organic cultivation—here meaning cultivation without the use of synthetic chemical inputs—dominated HDRA's early collective experiments and its publications. Most focused on how to control pests and diseases (e.g., blackfly, slugs, birds, and clubroot) via “natural” means and on the use of compost and green manure as fertilizers. Hills assembled a set of general recommendations on organic cultivation in a series of guides for gardeners in the 1960s and 1970s, including several editions of his *Grow Your Own Fruit and Vegetables*. Here, Hills (1971, 15–25) also made his case for why readers should grow their own produce using organic methods: “Own‐grown” (the term used by Hills) organic produce was cheaper and more nutritious, tasted better, and, most importantly, enabled individuals “to contract out” of the use of chemicals harmful to people and planet alike.

Although it was to become a central activity of the HDRA by the late 1970s, my review of available materials indicates that concerns about the loss of diversity in vegetable crops and the need for gardeners to save and exchange seed received no mention in HDRA publications of the 1960s and into the early 1970s. The 1960s saw two editions of Hills's “Good Taste Guide to Garden Fruit and Vegetables,” a pamphlet that listed what Hills and his HDRA member correspondents deemed “the best flavoured” varieties along with the seed companies and nurseries that stocked these varieties. It aimed to help would‐be buyers navigate a world where catalogs uselessly described every variety as having “a superb flavour, excellent cooking qualities and a colossal crop” (Hills 1969, 3). The pamphlet offered information on difficult‐to‐locate varieties and emphasized the superior qualities of many older varieties now neglected, but it did not express concern about their eventual disappearance or exhort readers to ensure their continued existence through seed saving. Nor did *Grow Your Own Fruit and Vegetables* (never substantially altered after the first edition in 1971) include instructions on seed saving among its 300‐plus pages of advice, instead directing growers to the best purveyors of seed and nursery stock.

It was the development of new regulations for the registration and sale of plant varieties in Britain that first led Hills, and HDRA members along with him, to begin worrying about the dwindling cultivation of diverse vegetable varieties and its potential consequences. In July 1973, the UK government, acting to align its policies with those required within the European Economic Community (EEC), began restricting the sale of seeds of particular crops to those varieties specified in either the newly published UK National List or the EEC Common Catalogue. Each of these registers was to represent the final outcome of an evaluation process by which the varieties sold to growers could be ensured to be distinct, uniform, and standard and to offer an advantage over others already registered and sold. Hills believed that the demands of the new National List, and especially the fine of £400 that would be levied against anyone who sold unregistered seed, would lead companies to abandon many varieties—and that those useful lines would be lost not only to growers in the near‐term but forever (Hills 1977, 1–2; Hills 1978, preface; also Gear and Gear 2009, 178–183). “Commercial pressure makes it impossible to list kinds that sell as few as a thousand packets a year, and the fine for selling varieties not on the national and EEC lists… prevents specialist seedsmen from stocking the Old Masters of the kitchen garden, or preserving their genes for the plant breeders of the future,” Hills (1975) explained in a letter to the *Times* of London.^5^

Although concerns about the consequences of the EEC list differentiate the history of seed saving at HDRA from its counterpart organizations in the U.S. (Schmidt 2015; Whealy 2011), references to other overarching worries suggest these histories still share much in common. Hills acknowledged more general threats to vegetable diversity, namely industrial agricultural production and the orientation of varietal development around its demands. For example, according to Hills, commercial British tomato growers could no longer afford to grow the old‐but‐delicious Market King variety due to its thin skin (which was prone to damage in processing) and low yields. In its stead, they had first embraced high‐yielding varieties despite their comparative tastelessness, then hybrid crosses, and “now the new Dutch kinds have triumphed until perhaps the majority of growers in Britain grow a single variety” (Hills 1977, 1). When growers stopped cultivating particular varieties, seed companies stopped maintaining these lines for sale, and unless some other organization stepped into ensure their continuation, these would not only go out of circulation but perhaps out of existence.

The changing regulatory and commercial context of the 1970s and growing national and international concerns about the need to conserve plant genetic resources created a new area of advocacy for Hills and HDRA. The organization, in turn, laid a new set of concerns and activities before its British own‐gardeners and scattered HDRA members worldwide. The organization's established advocacy of organic methods shared in an existing rhetoric around these methods that linked individual acts of cultivation with better stewardship of the Earth and its resources.^6^ Its new advocacy of vegetable variety diversity inscribed a further range of ordinary gardeners’ activities—purchasing certain kinds of seed, saving and exchanging seeds, and cultivating traditional varieties—with new meaning as urgent acts of conservation.

## The Vegetable Seed Bank

In his 1975 *Times* letter, Hills announced an initiative of HDRA intended to address these concerns: the collection of Europe's “vanishing vegetables.” Hills particularly had in mind the need to salvage varieties with better flavor and more useful traits, especially those appropriate to the kind of small‐scale, chemical‐free, subsistence cultivation his organization espoused. These included things such as winter‐hardy lettuces, fly‐resistant carrots, and varieties “superior in flavour” to contemporary releases. Hills assumed that these hardier, more flavorful varieties were mostly to be found in small towns and marginal areas (Hills 1975). His letters and HDRA publications initially encouraged any gardener in possession of treasured old varieties to write in with a history and description of these or to collect and submit catalogs of “seedsmen who specialise in old fashioned varieties” so that HDRA could search for and buy seeds of now‐endangered types (HDRA 1975a, 6; HDRA 1975b, 5). Later missives requested donations of any of “special varieties” not otherwise available (HDRA 1977, 18).

Collecting these endangered varieties was to be only the first step in their salvation. Simultaneous to launching the collecting effort, HDRA began a “campaign to start a Seed Bank” (Hills 1977, 2; see also HDRA 1975c, 6–9; HDRA 1978a, 33–40). Apparently convinced that what was needed to ensure the long‐term preservation of vegetable diversity was well beyond the current capacities of HDRA, Hills began searching for an organization that would back the creation of a vegetable seed repository. He envisioned that this operation would gather and preserve vegetable varieties from around the world, just as international organizations had launched collections of key crops such as wheat, maize, and rice (Curry 2017). Unlike those comparable seed banks, however, the ideal vegetable repository envisioned by Hills would function as both a “bank” and a “library.” The bank would be accessible only to HDRA staff and horticultural professionals and would preserve seed in long‐term cold storage facilities. Meanwhile, the seed library would remain open to all users regardless of professional status upon their payment of a subscription fee and would ensure that varieties remained in circulation and cultivation.^7^

Hills's campaign soon caught the attention of the chairman of the trustees of the British charity Oxfam, who, in turn, brought the idea to Brian Walker, Oxfam's Director General. Walker considered the vegetable seed bank a promising project, squarely in line with Oxfam's commitment to addressing food production in developing countries.^8^ In consultation with Hills, Walker convened international experts in the field of plant genetic resources and representatives from the UK Ministry of Agriculture, Fisheries and Food and UK Agricultural Research Council, which had already begun to consider national plant genetic conservation needs.^9^ These discussions eventually resulted in a successful Oxfam campaign for a Vegetable Gene Bank (VGB), which was established in 1980 as part of the existing UK National Vegetable Research Station at Wellesbourne, Warwickshire (Astley 1998).

The combined perspectives of the British agricultural research establishment, the international crop genetic conservation community, and the poverty‐relief charity Oxfam—and the imperatives arising through joint funding from these—produced an international vegetable seed collection housed within and overseen by a national research organization. The initial focus of the Vegetable Gene Bank was small‐seeded, temperate vegetables, such as onions, cabbages, and carrots. According to the organization's first director Dave Astley, this mission “reflected the national issues” that concerned the UK National Vegetable Research Station and the Ministry of Agriculture, Fisheries and Food, and “conveniently filled a gap” in the growing international network of crop seed collections (Astley 1998, 5).

The VGB was not the establishment Hills had envisioned. It looked very much like a hybrid between a national collection and one of the international crop gene banks established in the preceding decades for wheat, maize, and rice. Like these collections, the VGB was oriented toward the needs of professional plant breeders and was largely accessible only to these professionals. Despite its partial origins in the agitation of an inveterate champion of home gardening, the VGB would not serve the immediate needs of own‐growers. Although Hills had been involved in the development of the initial Oxfam campaign, he was not consulted in the design of the VGB, circumstances that apparently took him by surprise.^10^

## The Vegetable Finder

As the trajectory of the VGB became clear, Hills reoriented his campaigning toward the “library” component of his initial proposal; in the meantime, however, other activities at HDRA came into play. In 1977, HDRA published the first edition of *The Vegetable Finder*, a catalog of what Hills described as “the finest flavoured varieties, old and new” available from seed sellers via ordinary channels. Hills hoped that this booklet and a companion, *The Fruit Finder*, would encourage gardeners to order seeds (or plants and trees) of these varieties direct from commercial sellers, in turn convincing those sellers that it would be worth their while to keep the varieties registered on the National List. As he reported in his introduction to *The Vegetable Finder*, progress in the campaign for a seed bank had been slow, and thus, the aim of the booklet would be “to keep some of these fine old varieties in cultivation until an official International Vegetable Seed Bank can be established” (Hills 1977, 2).

Fifty pages long and 50 pence to purchase, *The Vegetable Finder* listed seed potato and vegetable varieties currently on the market that were deemed to be of high quality by HDRA. It also provided occasional growing advice, comprising (in most cases) a short description of the variety and (in all cases) a list of possible suppliers. The catalog descriptions indicate the characteristics that Hills and the two HDRA employees who compiled it, Alan and Jackie Gear, imagined their readers to be seeking in their vegetables. Consider the entries for three varieties of broad bean: The Seville Longpod had “shorter pods but more of them” and was “a better flavour variety.” The Sutton was “a short fat bean for windy gardens.” Meanwhile the Giant Four Seeded White Winsor produced “large beans that made Brown Windsor soup when canning was only a Prime Minister!” (Hills 1977, 14–15). Taste, hardiness, and tradition were the prized qualities that catalog entries highlighted.

*The Vegetable Finder* and *The Fruit Finder* had their roots in the earlier pamphlet created by Hills, the “Good Taste Guide to Garden Fruits and Vegetables.” However, this guide had aimed only to help gardeners acquire superior but less common varieties and made no explicit mention of the need to conserve them. EEC legislative changes in the 1970s, and concerns that these changes would drive some varieties to extinction, prompted the expansion of the “Good Taste Guide” into the *Finders* and their repurposing as tools of conservation. As *The Vegetable Finder* and *The Fruit Finder* reminded readers, purchasing old varieties was no longer just a route to better‐flavored own‐grown crops—it was essential to keeping them extant.

## The Vegetable Seed Library

While *The Vegetable Finder* was in preparation, HDRA continued the collecting effort launched in 1975, focusing on heirlooms and old commercial varieties no longer on the National List and therefore in danger of being lost. At first, Hills envisioned that these would be collected for safekeeping in the proposed international seed bank. When the bank was slow to develop and then took shape as a resource primarily for professional breeders, HDRA's collected seeds fed instead into the “HDRA Vegetable Seed Library for Research and Experiment,” which was launched officially in February 1978.

As the Gears later described, “During 1976 and 1977 we acquired as many seeds as possible. Some seedsmen gave us varieties they could no longer sell. Others were donated by members.” Miss Cutbush of Maidstone sent in what she described as seeds from “a very old broad bean variety, given to [her] father by a cottager many many years ago, a small, chunky, delicious seed” (HDRA 1978b, 42). The Bishop of Bath and Wells donated seeds of the Martock Bean, said to have been “grown continuously in the kitchen garden of the Bishop's Palace since the time of the Tudors” (Gear and Gear 2009, 182–183). The initial goal was to reproduce and share with HDRA members donations like these and other varieties identified as commercially unavailable. But this task was easily beyond the capacities of the small HDRA staff working their equally small farm. HDRA therefore recruited what it eventually referred to as “Seed Guardians,” members tasked with raising varieties for seed and donating this back to the organization for redistribution (Hills 1982).^11^

One significant hitch with the plan for HDRA to supply these unlisted varieties to British growers was that seed regulations made it illegal to sell them. HDRA could, however, give away seeds “for the purposes of experimentation,” just as seed companies did in order to test varieties in preparation for the official registration process. The organization began sharing its stocks in limited quantities to members free‐of‐charge (with a fee of 50 pence to cover postage), asking those who received seeds from these “forbidden varieties that have survived from the past” to keep information about growth habits, taste, and other characteristics (HDRA 1978a, 38–39; HDRA 1985, 25; Gear and Gear 2009, 183). HDRA anticipated that members’ experiments would “result in greater knowledge of [these varieties’] qualities which, in turn, will help preserve them from extinction” (HDRA 1978a, 39). In other words, gardeners’ informed assessments were considered as crucial to seed conservation as their physical labors.

As the arrangements for the creation and upkeep of the seed library were being put in place, HDRA also began to encourage individual seed saving. The 1975 booklet *Save Your Own Seed*, written by Hills, presented this activity first as a matter of economy and only second as an issue of keeping varieties in cultivation. By 1978, when HDRA published a second edition, priorities had shifted: “Three years ago… the major advantage of saving your own seed was the increasing cost of every packet. Inflation is still with us, but we now have a stronger reason still… the only way to enjoy the Goyas and Gainsboroughs of the kitchen garden, which are in greater danger than any wild flower, is to save your own seed” (Hills 1978, 1). It was not just HDRA or Seed Guardians whose labors contributed the larger project of keeping varieties extant, but any gardener who saved seed of the “Old Masters” of the kitchen garden.

As HDRA conservation activities proliferated beyond the initial agitation for an international seed bank, they focused on keeping varieties accessible as well as extant and emphasized the need for British gardeners’ active participation in order to achieve conservation goals. If the central purpose of *The Vegetable Finder* and *The Fruit Finder* was to keep varieties accessible by maintaining their profitability to sellers—therefore ensuring their continued registration and commercial availability—the seed library and individual acts of seed saving would ensure continued access to varieties that own‐growers valued but markets and regulatory agencies did not.

## Vegetable Sanctuaries

In addition to supplying the seed library with stocks for circulation to HDRA members, Seed Guardians also helped supply seeds to another set of operations launched by the HDRA with varied partners: vegetable sanctuaries. These were sites where property owners or managers promised to grow many endangered vegetable varieties to ensure that these continued to be exposed to changing environmental conditions (HDRA 1980a, 38; HDRA 1980b, 24–25; Hills 1982, 238). As with the seed library, HDRA publications offered a contrast between the work of a sanctuary and that of a seed bank. As one description suggested, “If we tuck away our heritage in Gene Banks and grow only the vegetable equivalents to Golden Delicious apples we shall never know if any of the hardier varieties from the past carry the genes for resistance, as an example, to the acid rain that is slaughtering European forests and poisoning Scottish lochs” (HDRA 1985, 25).

Gene banks were not the only explicit point of comparison for the vegetable sanctuaries among conservation institutions. The very designation of “sanctuary” pointed to the established role of wildlife preserves or sanctuaries in protecting endangered plant and animal species (see Hills 1984, 372). In addition, HDRA quickly settled on stately homes as the likeliest sites for sanctuaries in Britain, which coupled these sanctuaries to historic properties, an established area of conservation interest in Britain. Hills's comparison of older vegetable varieties to the “Old Masters” and HDRA's alliance with stately homes calls attention to the class and cultural underpinnings of the HDRA campaigns. Whereas comparable organizations in the U.S. typically used designations like “heirloom” and “traditional” to highlight family and community legacies (see Schmidt 2015; Whealy 2011), the HDRA's use of the term “heritage” linked vegetable seeds to the celebration and defense of British national heritage, and especially to institutions thought to reflect cultural refinement and even superiority.^13^

In the early 1980s, HDRA collaborated in the creation of several vegetable sanctuaries in the United Kingdom. The first, established in 1980, was at Dean's Court, a stately home in Wimborne, Dorset. Although Hills (1980) emphasized that its key contribution would be to enable the continuing evaluation of varieties, it also held the potential for public education. Other sites soon followed: Quarry Bank Mill in Cheshire (a National Trust site), Harlow Carr near Harrogate (then the gardens of the Northern Horticultural Society), and Croxteth Country Park near Liverpool (a publicly owned stately home and gardens). While the maintenance of these gardens remained the responsibility of the owners or keepers, HDRA provided the “historic seeds” that were to be, in Hills's words, “an attraction, like antique cars or lions” (Hills 1982, 238).

Vegetable sanctuaries (which Hills also hoped to establish in Europe and what he called the “Third World”) acknowledged a significant shortcoming of the genebanking system: Growers could not assess plants kept dormant in cold store under changing conditions. In Britain, sited on properties established as tourist spots or popular recreational spaces, sanctuaries also provided opportunities to draw public attention to the issue of declining vegetable diversity. Hills's frequent comparison of vegetable sanctuaries with wildlife reserves and cultural heritage preservation demonstrated his conviction that vegetable conservation was an activity of equivalent social importance. However, Hills and HDRA also emphasized that any garden where rare or threatened varieties were saved would be a sanctuary, as it would contribute to the work of perpetuating these endangered vegetables. In this sense, the possibility of managing a vegetable sanctuary was open to all HDRA members, and not just the keepers of a few stately homes.

## Conclusion

Within HDRA (as directed especially by Hills in this period), no single initiative was considered sufficient to secure vegetable diversity in the long term, nor could any one institution hope to succeed alone. HDRA descriptions of its vegetable conservation activities in the 1970s and 1980s often emphasized the interconnections between the labors of the organization, individual HDRA members, and professional research organizations and breeders. For example, HDRA emphasized that if participants in the vegetable seed library discovered “anything of interest to the Gene Bank” through their experiments and observations, this information would be “passed on and stored” (Hills 1982, 238). Vegetable sanctuaries would be sites for the preservation and study of the varieties of a particular region. However, they would also depend on outside expertise for technical assessments, such as vitamin content analysis and continued breeding. As Hills noted, “The knowledge that [vegetable sanctuaries] win, the programmes of breeding that this [knowledge] suggests and the germplasm material they can supply, will be of different value to government or commercial plant breeders” (Hills 1984, 373).

The commercial market was also an important element of the HDRA conservation vision. Prior to the creation of the Vegetable Gene Bank at Wellesbourne, Hills suggested that anything discovered through HDRA collecting and subsequent experimentation in the vegetable seed library would “be submitted to the Ministry of Fisheries and Food at Cambridge for testing prior to achieving National List Status.” This would usher some varieties from the under‐documented world of small‐scale cultivation and exchange into the world of national regulation and international commerce (Hills 1978, 3). HDRA hoped that commercial seed companies and nurseries could be made to appreciate the value of old varieties, not just as genetic source material to further develop established lines (a standard use of banked materials) but also as useful varieties in‐and‐of themselves. This assumed that a substantial enough market could be established for traditional varieties. HDRA's *The Vegetable Finder* and *The Fruit Finder* aimed at creating and sustaining just such a market.

National and international plant genetic conservation efforts in the 1970s coalesced around so‐called seed or gene banks as the preferred tool, but for those within HDRA, this was only one of many possible measures that could, and should, be deployed simultaneously. Although HDRA publications never questioned the importance of international gene banks as a conservation measure, they often emphasized their limitations: that they were accessible primarily to professional scientists and breeders, that they kept varieties as seed in cold store such that they could not be assessed in light of changing environments, and that the conditions of genebanking caused inevitable winnowing of genetic diversity. The HDRA seed library and a network of vegetable sanctuaries were needed to counteract these limitations, offering widened access to all growers and ensuring continuous cultivation at numerous sites with different prevailing ecological conditions.

One element among these many measures was crucial to their collective success: the commitment of British own‐gardeners to purchasing, growing, saving, and circulating seeds of useful or delicious varieties. Today, many home and allotment gardeners who save seeds see themselves as stewards or protectors of endangered plants and view their gardens as repositories of important biodiversity. In order for this perspective to have emerged, gardeners needed first to come to a new understanding of how ordinary gardening tasks ensured the very possibility of delicious, own‐grown harvests—for all growers and for many years to come. The diverse activities of HDRA contributed to the emergence of this conservation mindset among gardeners in Britain, where the organization played an early role in encouraging alternative farming and gardening methods and advocating for the importance of seed saving. Today, Garden Organic continues this legacy through the promotion of its Heritage Seed Library.

The history of HDRA's multiple and intersecting strategies for mobilizing own‐gardeners to cultivate and share prized heritage vegetable varieties offers a reminder of the important role played by membership organizations in shaping a shared idea of gardening as a political practice. More specifically, it points to the historically contingent trajectories that underlie a contemporary consensus about the social and ecological implications of individual seed saving. From the 1970s onward, HDRA encouraged British gardeners to reimagine seed saving as a conservation practice. Although the organization emerged from and responded to more general concerns about agricultural and environmental change, its promotion of seed saving had specific origins. HDRA campaigns for seed banks, sanctuaries, and libraries addressed the consequences of regulations that aimed to make European agriculture more efficient by limiting and standardizing the varieties offered to farmers—regulations whose ultimate consequence was the enforced scarcity of all other varieties. As a result of the HDRA's campaigns, own‐growers in Britain became partners in a specific project of preserving crop diversity, linked to but distinct from their existing efforts to protect land, wildlife, and human health through organic cultivation. By growing local favorites, British gardeners additionally preserved both national cultural heritage and the possibilities for diverse agricultural futures.

## Notes

1 For a valuable collection of such studies, see Nazarea, Rhoades, and Andrews‐Swann (2013). Here, I focus on the literature about seed savers in Europe and the U.S., as this scholarship is the most directly relevant for the present article. For studies of farmer‐saved seed elsewhere, useful starting points include Brush (2000, 2004).

2 “Own‐growers” is a term that Lawrence Hills sometimes used to describe gardeners raising vegetables and other crops for their own use. I prefer it to “home gardener” or “backyard gardener” in this account because of the prevalence of allotment gardening in Britain. Many HDRA members would have been growing vegetables for their own consumption (i.e., “own‐growing”) at a community site rather than their home or backyard.

3 Despite several attempts, I have been unable to locate organizational archives for HDRA or personal papers of its founder Lawrence Hills. My history is based primarily on surviving HDRA publications from this period, many of which are held at Garden Organic in Ryton‐on‐Dunsmore, United Kingdom.

4 The HDRA quarterly *Newsletter*, in which Hills typically offered a long letter to members and a digest of the observations and experimental outcomes he had received from them, is the best resource for understanding both the integration of members into the work of the organization and the leading role of Hills.

5 On the role of organizations in forging the links between organic cultivation and larger social and environmental outcomes, see Reed (2010), especially chapter 4.

6 Hills imagined other solutions to the accessibility problem, but this appears to have been the one he favored. HDRA, “Vegetable Seed Library, Preliminary Proposals,” May 1975, Archives of the Royal Botanic Gardens, Kew (hereafter, KEW), QEW 124.

7 Walker to Divisional Heads and Area Directors, 21 December 1976, Oxfam Archives: Communications, Bodleian Library, Oxford (hereafter, OA), COM 3‐1‐6, Folder 5.

8 Ibid.; [Walker], “Opening of the Wellesbourne Vegetable Gene Bank, 8 October 1980,” OA R9511‐2, Folder: Opening Ceremony Gene Bank, 2.

9 Hills's responses to the development of the VGB, including a new proposal for a Vegetable Seed Library in January 1976, can be found in KEW, QEW 124A.

10 HDRA organized the work of Seed Guardians so as to limit the possibilities of cross‐pollination among varieties and therefore maintain distinct lines. In the U.S., Seed Savers Exchange tested a similar system of volunteer curation a few years later, indicating that this was a shared hurdle for seed saving organizations that aimed to have a central collection in addition to a grower network (Seed Savers Exchange 1987, 93–96).

11 For analyses of the discourse of “heritage” in relation to fruit and vegetable varieties, see Jordan (2007) and Wincott (2015).
